# Reusable, set-based selection algorithm for matched control groups

**DOI:** 10.23889/ijpds.v1i1.395

**Published:** 2017-04-19

**Authors:** Daniel Thayer, John W Gregory, Liv Kosnes, Damon Berridge, Martin L Heaven, David V Ford, Keith Lloyd, Ann John

**Affiliations:** 1 Swansea University; 2 Cardiff University

## Aims

The wealth of data available in linked administrative datasets offers great potential for research, but researchers face methodological and computational challenges in data preparation, due to the size and complexity of the data. The creation of matched control groups in the Secure Anonymised Information Linkage (SAIL) Databank illustrates this point: SAIL contains multiple health datasets describing millions of individuals in Wales. The volume of data creates the potential for more precise matching, but only if an appropriate algorithm can be applied. We aimed to create such an algorithm for reuse by many research projects.

## Methods

We developed set-based code in SQL that efficiently selects matches from millions of potential combinations in a relational database environment. It is parameterized to allow different matching criteria to be employed as needed, including follow-up time around an index event. A combinatorial optimisation problem occurs when a potential control could match more than one subject, which we solved by ranking potential match pairs first by subject with the fewest potential matches, then by closeness of the match.

## Results

One example of the algorithm's use was the Suicide Information Database Cymru, an electronic case-control study on suicide in Wales between 2003 and 2011. Subjects who had a cause of death recorded as self-harm were each matched to twenty controls who were alive at the subject's date of death and had the same gender and similar birth week. The rate of matching success was >99.9%, with all subjects but one matching the full twenty controls. >99.99% of the matched controls had a week of birth that was identical to the subject. The second example was a matched cohort study looking at hospital admissions and type 1 diabetes, using the Brecon register of childhood diabetes in Wales, with matching based on week of birth within two weeks, gender, county of residence, deprivation quintile, and residence in Wales at time of diagnosis. This study had a matching rate of 98.9%; 97.5% of subjects matched to five controls, and 69.8% of matches had the same week of birth. 

## Conclusion

This algorithm provides good matching performance while executing efficiently and scalably on large datasets. Its implementation as reusable code will facilitate more efficient, high-quality research in SAIL. Instead of spending many hours developing a custom solution, analysts can execute parameterized code in a few minutes. We hope it to be useful more widely beyond SAIL as well.

